# MicroRNA-744 inhibits tumor cell proliferation and invasion of gastric cancer via targeting brain-derived neurotrophic factor

**DOI:** 10.3892/mmr.2022.12804

**Published:** 2022-07-27

**Authors:** Ai-Jun Xu, Li-Na Fu, Hua-Xing Wu, Xiao-Li Yao, Rui Meng

Mol Med Rep 16: 5055–5061, 2017; DOI: 10.3892/mmr.2017.7167

Following the publication of this paper, it was drawn to the Editors’ attention by a concerned reader that certain of the cell migration assay data shown in Fig. 2D were strikingly similar to data that had appeared in different form in other articles by different authors. Owing to the fact that the contentious data in the above article had already been published elsewhere, or were already under consideration for publication, prior to its submission to *Molecular Medicine Reports*, the Editor has decided that this paper should be retracted from the Journal. The authors were asked for an explanation to account for these concerns, but the Editorial Office did not receive a reply. The Editor apologizes to the readership for any inconvenience caused.

